# Validation of previously identified serum biomarkers for breast cancer with SELDI-TOF MS: a case control study

**DOI:** 10.1186/1755-8794-2-4

**Published:** 2009-01-19

**Authors:** Annemieke WJ van Winden, Marie-Christine W Gast, Jos H Beijnen, Emiel JTh Rutgers, Diederick E Grobbee, Petra HM Peeters, Carla H van Gils

**Affiliations:** 1 Julius Center for Health Sciences and Primary Care, University Medical Center Utrecht, Mail-drop Str 6.131, PO Box 85500, 3508 GA Utrecht, the Netherlands; 2 Department of Pharmacy & Pharmacology, the Netherlands Cancer Institute/Slotervaart Hospital, Amsterdam, the Netherlands; 3 Beta faculty, Department of Pharmaceutical Sciences, Division of Biomedical Analysis, Section of Drug Toxicology, Utrecht University, Utrecht, the Netherlands; 4 Department of Surgery, the Netherlands Cancer Institute/Antoni van Leeuwenhoek Hospital, Amsterdam, the Netherlands

## Abstract

**Background:**

Serum protein profiling seems promising for early detection of breast cancer. However, the approach is also criticized, partly because of difficulties in validating discriminatory proteins. This study's aim is to validate three proteins previously reported to be discriminative between breast cancer cases and healthy controls. These proteins had been identified as a fragment of inter-alpha trypsin inhibitor H4 (4.3 kDa), C-terminal-truncated form of C3a des arginine anaphylatoxin (8.1 kDa) and C3a des arginine anaphylatoxin (8.9 kDa).

**Methods:**

Serum protein profiles of 48 breast cancer patients and 48 healthy controls were analyzed with surface-enhanced laser desorption/ionization time-of-flight mass spectrometry (SELDI-TOF MS). Differences in protein intensity between breast cancer cases and controls were measured with the Mann-Whitney U test and adjusted for confounding in a multivariate logistic regression model.

**Results:**

Four peaks, with mass-to-charge ratio (*m/z*) 4276, 4292, 8129 and 8941, were found that were assumed to represent the previously reported proteins. *M/*z 4276 and 4292 were statistically significantly decreased in breast cancer cases compared to healthy controls (p < 0.001). M/*z *8941 was decreased in breast cancer cases (p < 0.001) and *m/z *8129 was not related with breast cancer (p = 0.87). Adjustment for sample preparation day, sample storage duration and age did not substantially alter results.

**Conclusion:**

*M/z *4276 and 4292 both represented the previously reported 4.3 kDa protein and were both decreased in breast cancer patients, which is in accordance with the results of most previous studies. *M/z *8129 was in contrast with previous studies not related with breast cancer. Remarkably, *m/z *8941 was decreased in breast cancer cases whereas in previous studies it was increased. Differences in patient populations and pre-analytical sample handling could have contributed to discrepancies. Further research is needed before we can conclude on the relevance of these proteins as breast cancer biomarkers.

## Background

In the search for new breast cancer biomarkers several studies have been performed comparing serum protein profiles of breast cancer cases with those of healthy controls using surface enhanced laser desorption/ionization time-of-flight mass spectrometry (SELDI-TOF MS) [1-5]. In these studies several proteins have been found to be linked with the presence of a breast tumor. However, only occasionally the same proteins are found to be associated with the disease state [1,4,5]. This is likely to be caused by the use of different protocols for sample handling, sample preparation and sample storage as well as the use of different ProteinChip arrays and binding- and wash buffers. However, even in validation studies using identical protocols, it has not been possible to replicate the results of previous studies entirely [2,4]. Differences in results may be caused by chance; simply because a large number of peaks is tested some proteins will be found to discriminate between breast cancer cases and healthy controls. Also differences between the patient populations (like in age) and in characteristics of the tumors may have led to different results.

For instance, in 2002 Li et al. [3] performed a study to identify serum biomarkers for invasive breast cancer. In this study three proteins with masses of 4.3 kDa, 8.1 kDa and 8.9 kDa were found that were together able to discriminate best between breast cancer cases and non-cancer controls. The non-cancer control group in this study consisted of both women with benign breast disease and healthy controls. The 4.3 kDa protein was decreased in breast cancer cases compared to non-cancer controls, the 8.1 and 8.9 kDa proteins were both increased [3].

In 2005, Mathelin et al. [2] performed a study to validate the results of Li et al. [3] with different samples in a different laboratory using the same assay. In this study five peaks were found which masses corresponded to those of the proteins found by Li et al. [2,3]. Two peaks probably representing the 4.3 kDa protein and its oxidized form (*m/z *(mass-to-charge ratio) of 4286 and 4302) were both statistically significantly decreased in breast cancer cases compared to non-cancer controls (women with benign breast disease and healthy controls). Two peaks that could possibly represent the 8.9 kDa protein (*m/z *of 8919 and 8961) were both statistically significantly increased in breast cancer cases compared to non-cancer controls [2]. Above mentioned findings are in accordance with those of Li et al. [3]. Contrary, the intensity of the peak likely to represent the 8.1 kDa protein (*m/z *of 8129) was not different between breast cancer cases and non-cancer controls in this study [2].

At the same time, Li et al. also performed a validation study [4] of the candidate biomarkers previously reported by their group. They used different samples but analyzed them in the same laboratory using the same assay. In this study women with benign breast disease were included as cases, together with women with ductal carcinoma in situ (DCIS) or stage I, II or III breast cancer. Three peaks with masses similar to those of the previously reported proteins were found to be discriminative between cases and controls. Strikingly, in this study the 4.3 kDa peak was increased in cases compared to controls, instead of decreased. The 4.3 kDa peak was identified in this study as a fragment of inter-alpha trypsin inhibitor heavy chain H4 (ITIH4). The 8.1 and 8.9 kDa peaks were again both increased in cases compared to controls and were identified in this study as a C-terminal-truncated form of C3a des arginine anaphylatoxin (C3a_desArgΔ88_) and C3a des arginine anaphylatoxin (C3a_desArg_), respectively [4].

Poteintial limitations of these studies include differences between cases and controls in storage duration [3] and age [2,3]. In the present study we analyzed samples of cases and controls, who were frequency matched for age at intake and storage duration of their serum sample, with the same protocol. With this study we aimed to determine whether the three previously reported proteins [2-4] are truly discriminative between breast cancer cases and healthy controls, after adjustment for any differences in age and storage duration.

## Methods

### Study population

We performed a case-control study with 48 women (aged 25 to 88 years) diagnosed with primary invasive breast cancer and 48 healthy controls (healthy female relatives or friends of the patients who accompanied them to the hospital). Cases and controls were frequency matched regarding their age and the storage duration of their serum sample as much as possible. Age and menopausal status of the cases were obtained through examination of the medical records. Tumor type, tumor size, tumor differentiation, lymph node involvement metastasis, oestrogen receptor (ER) and progesterone receptor (PR) status and HER2/neu and p53 expression were determined by pathological examination.

Serum samples of the cases and controls, which were collected between January 2003 and June 2005, were obtained from a serum bank at The Netherlands Cancer Institute (NKI), Amsterdam, The Netherlands. These serum samples were collected after receiving individuals' informed consent under approval of the Institutional Review Board Control. Serum samples of the cases were obtained after diagnosis of breast cancer and before surgery or any other kind of treatment. Blood collection, processing and storage of the serum samples was performed under strictly defined conditions which were the same for cases and controls. All serum samples were collected with the use of BD Vacutainer SST plastic serum tubes with clot activator and gel (Becton-Dickinson, Franklin Lakes, NJ, USA). After collection, blood samples were allowed to clot for 30 minutes at room temperature and were subsequently centrifuged for 15 minutes at 3000 rpm at room temperature. Thereafter, samples were aliquoted and stored at -30°C.

### Protein profiling

We executed the same sample preparation protocol and used the same ProteinChip arrays (IMAC30 activated with nickel; Bio-Rad Laboratories, Hercules, Ca, USA) and wash- and binding buffers as described by Li et al. [3]. A minor difference is that in this study unsaturated instead of saturated sinapinic acid (SPA; Bio-Rad Laboratories) was used, prepared according to manufacturer's instruction.

Samples of breast cancer cases and controls were alternately and in duplicate manually applied to the arrays. Half of the case samples and half of the control samples were prepared and applied to the array on day 1 and the other half of the case and control samples were prepared and applied to the array on day 2. Detection of the proteins bound to the arrays was performed with SELDI-TOF MS for all arrays on the same day. For this we used the newest SELDI-TOF MS instrument, the PCS 4000 ProteinChip Reader, the enterprise edition (Bio-Rad Laboratories). Contrary to the first generation instruments, the PCS 4000 has an increased dynamic range of the detector. This means that it has no fixed maximal signal and therefore saturation of the detector is less likely to occur. Furthermore, instead of using arbitrary units, peak intensities are scaled in μA, corresponding to the real electric current generated by the impact of ions onto the detector [6].

At the ProteinChip Reader, 10 shots with an intensity of 4500 nJ were fired on every fourth position of the entire spot. The detector attenuation was set to 1000 Da and masses up to 200,000 Da were detected with a focus at 8000 Da. The *m/z *was calibrated externally with an All-in-1 standard peptide mixture containing vasopression (1084.3 Da), somatostatin (1637.9 Da), dynorphin (2147.5 Da), ACTH 1–24 (2933.5 Da), bovine insulin beta-chain (3495.9 Da), human insulin (5807.7 Da) and recombinant hirudin (6963.5 Da) (Bio-Rad Laboratories). Data were analyzed with the ProteinChip Software package, version 3.1 (Bio-Rad Laboratories). Baseline subtraction was applied to the spectra and intensities of masses between 3,000 and 200,000 Da were normalized to the average total ion current (TIC) of all spectra. Ion noise from the matrix passed through in the spectra up to 3,000 Da and therefore this region was excluded from the analysis. Spectra with a very high or low TIC were eliminated from the analysis. This was the case if the normalization factor (NF) of a spectrum deviated more than two standard deviations (SD) from the mean NF.

With the Biomarker Wizard (BMW) software application (Bio-Rad Laboratories), peaks with a signal-to-noise ratio greater than 5 and which were present in at least 20% of the spectra were auto-detected. Peak clusters were completed with peaks with a signal-to-noise ratio greater than 2, within a 0.3% mass window of the detected peaks. In the spectra with no peak in a detected peak cluster, a mark was placed at the average *m/z *of that peak cluster. In the duplicate spectra of a subject, the intensities of the peaks with the same mass were averaged. To estimate the reproducibility of these duplicates the median coefficient of variance (CV) and the inter-quartile range (IQR) was calculated per *m/z *in cases and controls together.

### Data analysis

The Mann-Whitney U test was used to test if the median intensities of the detected peaks were statistically significantly different (p-value < 0.01) between breast cancer cases and healthy controls. The area under the ROC (Receiver Operating Characteristic) curve (AUC) was estimated per peak to evaluate the performance of a peak to classify samples into the two groups.

The peaks with an *m/z *most similar to the mass of the previously reported proteins [2-4] were selected and all analyses described below were performed on these peaks. These peaks were most likely to represent the previously reported proteins [2-4], also because these peaks were detected under the same conditions as were used to detect the previously reported proteins [2-4]. We analyzed the same matrix (serum) on the same array type using the same protocol. By doing this we did not only select those proteins with the same mass, but also those binding under the same conditions to the chip, indicating similarity in pI.

In the control group we investigated whether age, the duration of sample storage and day of sample preparation influenced peak intensity. For this, we compared the median intensities of the peaks between different categories of these variables with the Kruskal-Wallis test (>2 categories) or with the Mann-Whitney U test (2 categories). To this end, controls were categorized according to tertiles of age; < 49.3 years, 49.3 – 57.5 years or > 57.5 years and were also categorized according to quartiles of sample storage duration; < 12 months, 12 – 17 months, 18 – 31 months or > 31 months. Controls were also divided in two groups according to day of sample preparation; day 1 or day 2. Subsequently, the Jonckheere-Terpstra test was used to test if there was a trend in the median peak intensities between the different categories of age and sample storage duration.

To investigate whether any relationship between the intensity of the peaks and the presence of a breast tumor could be explained by above-mentioned variables, a logistic regression analysis was performed, estimating the crude odds ratio (OR) and the OR adjusted for these variables. For this purpose cases and controls were categorized by tertiles of peak intensity (low, intermediate or high intensity), based on the distribution in the control group. Because the range in peak intensities strongly differed between samples prepared on day 1 and those prepared on day 2, this was done separately for the set of cases and controls prepared on day 1 and the set prepared on day 2. Afterwards, the subjects in the same category in the two sets were combined. We also performed a backward logistic regression analysis in which we simultaneously included the peaks assumed to represent the previously reported proteins [2-4] (continuous). Peaks were removed from the model if they did not statistically significantly contribute to the discrimination of cases and controls.

To investigate whether the intensities of the peaks were related to stage of disease, we tested if the median peak intensities were different between categories of TNM stage, tumor size, lymph node involvement and tumor differentiation. We also tested if the peak intensities were related to menopause status, hormone receptor status and HER2/neu and p53 expression. The relation between menopause status and peak intensities was investigated in cases only, since no information about this variable was available for the controls. To test these relations we used the Kruskal-Wallis test or the Mann-Whitney U test, dependent on number of categories. TNM stage was categorized as: IIA (*n *= 26), IIB (*n *= 11) or III (= IIIA + IIIC, *n *= 11) and tumor size as: = 2 cm (*n *= 18) or > 2 cm (*n *= 30). Lymph node involvement was categorized as: no regional lymph node metastasis (*n *= 9) or positive axillary lymph nodes (*n *= 39). Tumor differentiation was classified into high and intermediate differentiation (*n *= 25) or low differentiation (*n *= 22) (1 missing value). Menopause status was categorized as pre- (*n *= 15) or postmenopausal (*n *= 30) (2 missing values). ER and PR status were categorized as ER- (*n *= 12) or ER+ (*n *= 35) and PR- (*n *= 19) or PR+ (*n *= 28). HER2/neu and p53 expression was categorized as HER2/neu- (*n *= 35) or HER2/neu+ (*n *= 12) and p53- (n = 18) or p53+ (*n *= 23) (6 missing values). The Jonckheere-Terpstra test was used to test if there was a trend in the median peak intensities between the different categories of TNM stage. For above mentioned statistical analyses SPSS 12.0.1 was used and p-values < 0.05 were considered statistically significant.

## Results

### Study population

The characteristics of the breast cancer cases and healthy controls are presented in Table 1. The average age at time of blood collection was 58 years for the cases and 53 years for the controls. Sixty-three percent of the cases was postmenopausal and 33% was premenopausal. The menopausal status of two women was not reported in their medical record. No information about menopause status was available for the controls. For cases, the median duration between diagnosis and sample collection was 7 days and all samples were collected before the start of treatment. The median duration of sample storage until analysis was almost equal for the samples of the cases (16 months) and those of the controls (17 months).

**Table 1:** Characteristics of the breast cancer cases, the healthy controls and their serum samples

	**Breast cancer cases** (*n *= 48)	**Healthy controls** (*n *= 48)
**Age **(years)		
Mean (± SD)	58 (± 14)	53 (± 9)

**Menopause status**, *n *(%)		
Pre	16 (33)	-
Post	30 (63)	-
Missing	2 (4)	48 (100)

**Sample storage duration **(months)		
Median (IQR)	16 (11–35)	17 (11–31)

**Time from diagnosis to blood sampling **(days)		
Median (IQR)	7 (0–20)	

**Stage**, *n *(%)		
IIA	26 (54)	
IIB	11 (23)	
IIIA	6 (13)	
IIIC	5 (10)	

**Tumor size**, *n *(%)		
> 0.5 – 1 cm	2 (4)	
> 1 – 2 cm	16 (33)	
> 2 – 5 cm	28 (58)	
> 5 cm	2 (4)	

**Lymph node involvement**, *n *(%)		
No	9 (19)	
1–3	29 (60)	
> 3	10 (21)	

**Differentiation**, *n *(%)		
High	6 (13)	
Intermediate	19 (40)	
Low	22 (46)	
Unknown	1 (2)	

**ER status**, *n *(%)		
Negative	13 (27)	
Positive	35 (73)	

**PR status**, *n *(%)		
Negative	20 (42)	
Positive	28 (58)	

**Her2/neu expression**, *n *(%)		
Negative	36 (75)	
Positive	12 (25)	

**P53 expression**, *n *(%)		
Negative	18 (38)	
Positive	24 (50)	
Missing	6 (13)	

SD = Standard Deviation, IQR = Inter-Quartile Range, ER = Oestrogen Receptor, PR = Progesterone Receptor

More than half of the cases was affected with stage IIA breast cancer and nearly a quarter of the cases was diagnosed with stage IIB breast cancer. Stage III breast cancer was diagnosed in the other cases. More than 60% of the tumors were larger than 2 cm and in more than 80% of the cases the tumor had spread to the axillary lymph nodes. Eighty-six percent of the tumors showed a low or intermediate degree of differentiation (1 missing value). None of the cases was diagnosed with distant metastases. The majority of the tumors was ER+ (73%) and PR+ (58%). A quarter of the tumors showed an overexpression of HER2/neu and half of the tumors had an overexpression of p53. For 6 cases (13%) p53 status was unknown. These subjects were diagnosed in The Netherlands Cancer Institute (NKI), however their surgery was performed in another hospital. In those hospitals, the p53 status of the tumor was not determined.

### Peak detection

After normalization, 17 of the 192 spectra (48 cases and 48 controls in duplicate) were eliminated from the analysis because their NF deviated more than two SD from the mean NF. These spectra belonged to 10 controls and 6 cases. Of one case both spectra (both duplicates) had to be eliminated. With the BMW software application, 45 peak clusters were auto-detected in the 175 left spectra, in the mass-region between 3,000 Da and 200,000 Da. Subsequently, in the duplicate spectra of a subject, the intensities of the detected peaks with the same mass were averaged. For the subjects with one spectrum left, only peak intensities in that spectrum were used for analysis. A Mann-Whitney U test performed on these (averaged) peak intensities showed that the intensities of 20 of the 45 peaks were statistically significantly different between breast cancer cases and healthy controls (p-value < 0.01). These discriminatory peaks are listed in a table in Additional file 1 in order of *m/z*.

Four peaks were detected which we assumed to represent the three previously reported proteins [2-4] based on similarities in mass and detected under the same conditions. The median intensity of these peaks, as well as the p-value of the Mann-Whitney U test and the AUCs are listed in Table 2 in order of the *m/z *of the peaks. Two peaks were detected that both were assumed to represent the previously reported protein of 4.3 kDa [2-4]. This protein was identified by Li et al. [4] as a fragment of ITIH4 and has a theoretical molecular weight (Mw) of 4285 Da. We based this mass on the amino acid sequence reported by Song et al. [7] and calculated it using ExPASy Proteomic Server [8]. The two peaks found in our study had an *m/z *which was almost similar to that Mw, namely 4276 and 4292. The mass difference between these two peaks is 16 Da, the exact mass of an oxygen-atom. The intensities of these two peaks were also highly correlated (Pearson R^2 ^= 0.834; p < 0.001 (in the control group)), indicating that they were present in about the same ratio in every spectra. Consequently, we assumed *m/z *4276 to be the 4.3 kDa ITIH4 fragment and m/z 4292 to be the oxidized form of this protein. Both these peaks should therefore be considered for the comparison with the previously reported 4.3 kDa protein [2-4]. The median intensity of both peaks was statistically significantly decreased in cases compared to controls (11.04 [IQR: 2.10–29.31] versus 39.48 [IQR: 14.17–77.58]; p < 0.0001 and 14.54 [IQR: 8.52–29.54] versus 42.07 [IQR: 29.67–63.39]; p < 0.0001, respectively).

**Table 2:** Intensities in cases and controls of the peaks most likely representing the previously reported proteins [2-4] in order of *m/z*

	**Breast cancer cases** (*n *= 47)		**Healthy controls** (*n *= 48)			**Mann-Whitney U test**	**ROC-curve**	
*M/z*	*Median intensity*	*IQR*	*Median* *intensity*	*IQR*	*Intensity in cases vs. controls*	*p-value*	*AUC*	*95%CI*

**4276**	11.04	2.10 – 29.31	39.48	14.17 – 77.58	Decreased	<0.0001	0.716	0.61–0.82
**4292**	14.54	8.52 – 29.54	42.07	29.67 – 63.39	Decreased	<0.0001	0.770	0.67–0.87
**8129**	25.92	20.48 – 28.19	26.05	21.06 – 28.08	-	0.870	0.510	0.39–0.63
**8941**	31.27	24.07 – 44.88	73.47	48.69 – 86.72	Decreased	<0.0001	0.830	0.75–0.91

*M/z *= Mass to Charge ratio, IQR = Inter-Quartile Range, ROC-curve = Receiver Operating Characteristic curve, AUC = Area Under the Curve, 95%CI = 95% Confidence Interval

One peak was found in this study that was assumed to represent the previously reported protein of 8.1 kDa [2-4]. This protein was identified by Li et al. [4] as a C-terminal-truncated form of C3a_desArg_. Its theoretical Mw is 8133 Da, based on the amino acid sequence reported by Li et al. [4] and calculated using ExPASy Proteomic Server [8]. The *m/z *of the peak found in our study was almost identical to that Mw, namely 8129. However, no difference in the intensity of this peak was observed between cases and controls (p = 0.87).

**Figure 1:**
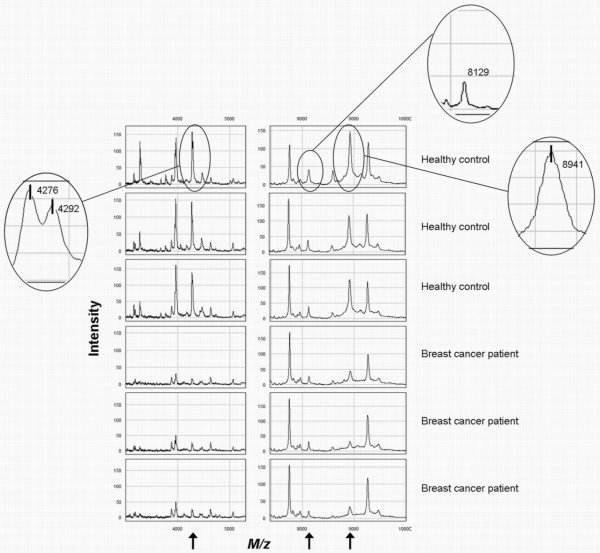
**Representative SELDI-TOF MS spectra showing the intensity of the peaks with an *m/z *of 4276, 4292, 8129 and 8941 in breast cancer cases and healthy controls**.

Also one peak was found that was assumed to represent the previously reported protein of 8.9 kDa [2-4]. This protein was identified by Li et al. [4] as C3a_desArg _and has a theoretical Mw of 8938 Da. We based this mass on the amino acid sequence reported by Li et al. [4] and calculated it using ExPASy Proteomic Server [8]. The *m/z *of the peak found in our study was almost identical to that Mw, namely 8941. The intensity of this peak was statistically significantly decreased in cases compared to controls (31.27 [IQR: 24.07–44.88] versus 73.47 [IQR: 48.69–86.72]; p < 0.0001). The small mass differences between the theoretical masses of the previously found proteins and the *m/z *of the peaks found in our study could be due to the mass calibration of the ProteinChip Reader used. Representative spectra from breast cancer cases and healthy controls showing the four peaks found in this study are presented in Fig. 1.

The median CV's per peak in cases and controls were 31% [IQR: 15–62], 17% [IQR: 7–47], 11% [IQR: 5–22] and 13% [IQR: 5–23] for *m/z *4276, 4292, 8129 and 8941, respectively. The duplicate analyses were also investigated separately and the results for the four peaks in the two analyses were similar to each other and to the results of the total analysis, i.e. the difference in intensity between cases and controls was similar with comparable significance levels (data not shown).

### Influence of day of sample preparation, sample storage duration and age on peak intensities

The relations between peak intensities and day of sample preparation, sample storage duration and age were estimated in the control group. The results are presented in Table 3.

**Table 3:** Intensities of the peaks per day of sample preparation, category of sample storage duration and age group in healthy controls

		***M/z *4276**	***M/z *4292**	***M/z *8129**	***M/z *8941**
	*n*	*Median intensity (IQR)*	*Median intensity (IQR)*	*Median intensity (IQR)*	*Median intensity (IQR)*

**Day of sample preparation**					
Day 1	24	74.04 (47.56–103.27)	62.78 (45.17–79.91)	27.39 (22.79–29.62)	78.94 (45.96–84.49)
Day 2	24	24.83 (9.58–38.24)	34.61 (28.54–39.32)	22.65 (18.02–26.89)	72.85 (50.43–105.05)
p-value*	48	<0.001	0.001	0.008	0.853

**Sample storage** **duration **(months)					
≤ 11	13	75.81 (33.59–118.06)	63.48 (37.10–79.58)	27.54 (21.94–31.63)	57.48 (43.11–79.66)
12–17	11	72.28 (50.60–87.56)	62.11 (46.25–83.88)	27.24 (24.70–29.87)	81.30 (52.43–99.14)
18–31	12	32.33 (12.37–40.50)	33.99 (25.21–45.65)	23.66 (15.58–27.08)	62.97 (42.67–111.06)
≥ 32	12	16.43 (8.38–35.85)	34.77 (29.84–37.99)	22.18 (20.46–26.89)	73.47 (52.76–105.05)
p-value^#^	48	0.001	0.011	0.063	0.620
p-trend^†^	48	<0.001	0.003	0.032	0.423

**Age **(yrs)					
< 49.3	16	45.26 (19.00–77.58)	46.38 (31.02–62.95)	25.15 (21.29–27.55)	73.84 (49.50–84.49)
49.3–57.5	16	19.79 (8.38–62.11)	35.78 (28.52–63.14)	24.90 (21.98–27.62)	69.16 (39.17–85.8)
> 57.5	16	45.90 (23.58–85.81)	40.01 (30.47–72.05)	27.12 (20.43–33.59)	76.15 (56.15–101.64)
p-value^#^	48	0.346	0.680	0.753	0.850
p-trend^†^	48	0.583	0.940	0.438	0.880

*M/z *= Mass to Charge ratio, IQR = Inter-Quartile Range, * Mann-Whitney U Test, ^# ^Kruskal Wallis Test, ^† ^Jonckheere-Terpstra Test

A clear relation between peak intensity and day of sample preparation was found for the peaks with an *m/z *of 4276, 4292 and 8129. The intensities of these peaks were statistically significantly higher in control samples prepared on day 1 than those prepared on day 2. We also found a statistically significant trend in intensity over the four storage duration categories for these three peaks, with lower intensities in samples that were stored for a longer time. Unintentionally, samples stored for less than 18 months were all prepared on day 1 and samples stored for 18 months or more on day 2. Therefore, it is difficult to disentangle the effects of day of preparation and storage duration. Age was not significantly related to the intensity of any of the peaks as shown in Table 3.

### Relationships between peak intensities and the presence of breast cancer

The relationships between the intensities of the four peaks and the presence of breast cancer, before and after adjustment for day of sample preparation, sample storage duration and age are listed in table 4. For the peaks with an *m/z *of 4276, 4292 and 8941, women with a low peak intensity were statistically significantly more often affected with breast cancer than women with a high peak intensity (reference group). The crude OR's with 95% CI (confidence interval) for these three peaks were 6.0 [95% CI: 2.0–18.2], 7.6 [95% CI: 2.4–24.3] and 13.0 [95% CI: 3.3–50.8], respectively. Women with an intermediate peak intensity for any of these three peaks were not more likely to be affected with breast cancer than women in the reference group. The intensity of the peak with an *m/z *of 8129 was not related to the presence of breast cancer. After adjustment for day of sample preparation, sample storage duration and age results remained essentially the same.

**Table 4:** The relationships between peak intensities and the presence of breast cancer before and after adjustment for sample characteristics and subject age

	***n***		**Crude OR **(95%CI)	Adjusted OR* (95%CI)
	*cases*	*controls*		

***M/z *4276**				
Low intensity	36	16	6.0 (2.0–18.2)	5.3 (1.7–17.0)
Intermediate intensity	5	16	0.8 (0.2–3.3)	0.9 (0.2–3.7)
High intensity	6	16	1.0 (referent)	1.0 (referent)

***M/z *4292**				
Low intensity	38	16	7.6 (2.4–24.3)	6.4 (1.8–22.3)
Intermediate intensity	4	16	0.8 (0.2–3.5)	0.7 (0.2–3.1)
High intensity	5	16	1.0 (referent)	1.0 (referent)

***M/z *8129**				
Low intensity	13	16	0.8 (0.3–2.3)	0.7 (0.2–2.0)
Intermediate intensity	19	17	1.1 (0.4–2.9)	1.0 (0.4–2.8)
High intensity	15	15	1.0 (referent)	1.0 (referent)

***M/z *8941**				
Low intensity	39	16	13.0 (3.3–50.8)	13.3 (3.2–55.0)
Intermediate intensity	5	16	1.7 (0.3–8.2)	1.8 (0.4–9.1)
High intensity	3	16	1.0 (referent)	1.0 (referent)

* OR's (odds ratios) with 95%CI (95% Confidence Interval) were adjusted for the following variables; day of preparation (day 1 or day 2), age (continuous) and storage duration (continuous).

*M/z *= Mass to Charge ratio, Tertiles of the intensities per peak were determined in the controls, separately for day of preparation, applied to cases prepared on the same day and afterwards combined in three categories.

Subsequently, we performed a backward logistic regression analysis in which we simultaneously included the four peaks (continuous). *M/z *8129 did not statistically significantly contribute to the model. *M/z *4276, *m/z *4292 and *m/z *8941 all contributed statistically significantly to the discrimination of cases and controls (data not shown).

### Relationships between peak intensities and tumor characteristics and menopause status

The relations between the intensities of the four peaks and tumor stage, tumor size, lymph node involvement and tumor differentiation are shown in Table 5. The median intensities of the peaks with an *m/z *of 4276 and 4292 in cases without lymph node involvement were higher than in cases with lymph node involvement (*m/z *4276: 24.34 [IQR: 5.99–72.28] versus 8.66 [IQR: 1.87–24.37] and *m/z *4292: 25.69 [IQR: 9.91–54.35] versus 13.13 [IQR: 5.97–23.74], although not statistically significantly (p = 0.14 and p = 0.14). No statistically significantly relations were observed between the intensities of the four peaks and any of the other tumor characteristics.

**Table 5:** Intensities of the peaks for different categories of the tumor characteristics

		***M/z *4276**	***M/z *4292**	***M/z *8129**	***M/z *8941**
	*n*	*Median intensity* *(IQR)*	*Median intensity* *(IQR)*	*Median intensity* *(IQR)*	*Median intensity* *(IQR)*

**TNM stage**					
IIA	26	13.00 (1.79–83.10)	19.51 (8.07–54.23)	26.27 (23.48–30.07)	33.56 (24.09–50.43)
IIB	10	4.97 (1.33–17.24)	11.44 (4.83–15.79)	25.44 (16.56–27.75)	30.49 (20.16–34.59)
III	11	11.88 (4.89–24.69)	15.86 (9.88–22.87)	25.70 (15.68–26.54)	37.55 (25.26–47.29)
p-value*	47	.340	.211	.203	.473
p-trend^#^	47	.553	.368	.075	.667

**Tumor size**					
0.5–2 cm	18	10.24 (1.36–85.58)	18.44 (5.44–63.09)	26.53 (22.86–30.08)	30.48 (23.24–61.21)
> 2 cm	29	11.88 (3.97–24.51)	12.21 (8.71–24.28)	25.90 (19.08–27.45)	32.90 (24.04–44.16)
p-value^†^	47	.844	.526	.204	.896

**Lymph node involvement**					
No	9	24.34 (5.99–72.28)	25.69 (9.91–54.35)	26.48 (23.33–30.29)	38.92 (27.68–53.99)
Yes	38	8.66 (1.87–24.37)	13.13 (5.97–23.74)	25.81 (18.87–27.65)	30.49 (23.30–43.31)
p-value^†^	47	.137	.144	.224	.224

**Differentiation**					
High-Intermediate	25	11.04 (2.99–24.47)	14.06 (7.86–22.52)	25.72 (19.84–27.45)	31.27 (24.13–42.65)
Low	21	19.90 (2.98–60.12)	17.06 (8.71–44.18)	26.54 (19.68–29.79)	28.99 (23.34–45.88)
p-value^†^	46	.635	.384	.360	.903

*M/z *= Mass to Charge ratio, IQR = Inter-Quartile Range, * Kruskal Wallis Test, ^# ^Jonckheere-Terpstra Test, ^† ^Mann-Whitney U Test

The relations between the intensities of the four peaks and menopause status, hormone receptor status and HER2/neu and p53 expression are listed in Table 6. For *m/z *8129, a statistically significantly decrease in intensity was observed in postmenopausal cases compared to premenopausal cases (25.3 [IQR: 17.8–26.6] versus 27.6 [IQR: 22.2–32.7]; p = 0.03). In cases with p53– tumors compared to cases with p53+ tumors, the decrease in intensity for *m/z *8129 was borderline statistically significantly (23.7 [IQR: 17.6–26.8] versus 26.5 [IQR: 24.8–30.1]; p = 0.07). No other statistically significant relation was found.

**Table 6:** Intensities of the peaks for different categories of hormone-receptor status, Her2/neu and P53 expression and menopause status

		***M/z *4276**	***M/z *4292**	***M/z *8129**	***M/z *8941**
	*n*	*Median intensity* *(IQR)*	*Median intensity* *(IQR)*	*Median intensity* *(IQR)*	*Median intensity* *(IQR)*

**ER status**					
Negative	12	15.2 (0.7–70.6)	18.3 (6.2–48.1)	26.3 (25.2–28.5)	39.9 (24.3–57.5)
Positive	35	11.0 (3.9–24.3)	14.5 (8.9–22.9)	25.7 (17.9–28.2)	30.5 (24.1–43.4)
p-value*	47	.961	.696	.421	.380

**PR status**					
Negative	19	7.1 (4.0–29.3)	10.9 (8.5–30.8)	26.0 (21.7–27.8)	31.3 (24.0–43.3)
Positive	28	14.6 (1.8–28.2)	15.4 (6.8–28.7)	25.8 (19.5–29.6)	32.1 (24.1–46.7)
p-value*	47	.649	.696	.762	.588

**Her2/neu expression**					
Negative	35	11.0 (1.9–29.3)	15.9 (5.6–29.5)	25.9 (20.5–27.8)	32.9 (24.1–46.9)
Positive	12	9.5 (4.3–66.2)	11.1 (9.1–43.2)	26.0 (16.2–29.3)	29.7 (23.3–41.7)
p-value*	47	.961	.826	.751	.575

**P53 expression**					
Negative	18	6.5 (1.6–24.3)	12.7 (5.3–31.3)	23.7 (17.6–26.8)	27.1 (17.7–42.6)
Positive	23	11.0 (4.7–29.4)	14.9 (8.9–29.5)	26.5 (24.8–30.1)	32.9 (27.8–60.8)
p-value*	41	.599	.546	.070	.172

**Menopause status**					
Premenopausal	15	7.9 (1.9– 84.3)	12.2 (10.1–69.2)	27.6 (22.2–32.7)	29.0 (22.7–43.3)
Postmenopausal	30	10.2 (2.0–25.6)	14.3 (5.5–29.8)	25.3 (17.8–26.6)	32.1 (24.2–45.4)
p-value*	45	.942	.580	.030	.563

*M/z *= Mass to Charge ratio, IQR = Inter-Quartile Range, * Mann-Whitney U Test, ER = Oestrogen Receptor, PR = Progesterone Receptor

### Protein identity

The assumption that the proteins found in this study indeed represent the previously reported proteins [2-4] was based on similarities in the mass as well as on the conditions under which these proteins were detected. However, there is also another indication to assume that *m/z *4276 and *m/z *4292 indeed represent the 4.3 kDa fragment of the ITIH4 protein and its oxidized form. We found three other peaks in this study which masses (*m/z *3156, *m/z *3270 and *m/z *3965) highly corresponded with the theoretical masses of three other fragments of ITIH4 previously described by Villanueva et al. [9] and Song et al. [7] (3158 Da, 3274 Da and 3972 Da). We based these theoretical masses on the amino acid sequences reported by Villanueva et al. [9] and Song et al. [7] and calculated them using ExPASy Proteomic Server [8]. The intensities of *m/z *3270 and *m/z *3965 were highly correlated with the intensities of *m/z *4276 and m/z 4292 (*m/z *3270 and *m/z *4276: R^2 ^= 0.812, *m/z *3270 and *m/z *4292: R^2 ^= 0.722, *m/z *3965 and *m/z *4276: R^2 ^= 0.821 and *m/z *3965 and *m/z *4292: R^2 ^= 0.756 [p < 0.001 for all]). This high correlation in intensity is only expected when all these proteins originated from the same protein. Since masses of all these proteins have very high resemblances with the theoretical masses of the previously reported ITIH4 fragments and these proteins were all detected under the same conditions, we can assume that all these peaks represent fragments of ITIH4.

The peak with an *m/z *of 8941 was actually previously identified in our laboratory in a breast cancer serum sample as C3a_desArg_. The method of protein identification was similar to that performed in the validation study by Li et al. [4]. The 8.9 kDa protein was purified using QhyperD fractionation (Biosepra Inc., Malborough, MA, USA) and concentrated on YM50 spin concentrators (Millipore, Billerica, MA, USA). Subsequently, the eluate with the 8.9 kDa protein was de-salted on RP18 beads (Bio-Rad Laboratories). This purification process was monitored by profiling each fraction on IMAC30 Ni arrays and NP20 arrays (a non-selective, silica chromatographic surface) (Bio-Rad Laboratories). The de-salted eluate containing the 8.9 kDa protein was subsequently subjected to SDS-PAGE analysis. Gel electrophoresis was performed on Novex NuPage gels (18% Tris-Glycine gel; Invitrogen, San Diego, CA, USA). After staining, the band in the 8.9 kDa region was excised and subjected to passive elution followed by tryptic digestion of the eluate. Profiling of the gel-eluate on a NP20 array confirmed the presence of the 8.9 kDa protein. Peptide mapping of the tryptic digest identified it as complement component 3 precursor (estimated Z-score 1.57, 4% sequence coverage). Amino acid sequencing of 6 peptides in the tryptic digest by tandem MS on a Q-TOF identified the protein as C3a des-arginine anaphylatoxin (C3a_desArg_, 61% sequence coverage), a protein with theoretical mass 8939.46 Da and pI 9.54. This identity was confirmed by an immunoassay, for which ProteinA beads were loaded with a C3a polyclonal antibody (Abcam Ltd, Cambridge, UK).

## Discussion

In this study including 48 breast cancer cases and 48 healthy controls we discovered four peaks with an *m/z *(4276, 4292, 8129 and 8941) within the mass range of the three previously reported proteins (4.3 kDa, 8.1 kDa and 8.9 kDa) [2-4] using a similar analysis protocol [3]. Three of these four peaks were found to be discriminative between breast cancer cases and healthy controls. The peaks with an *m/z *of 4276, 4292 and 8941 were all statistically significantly decreased in cases compared to controls. The intensity of the peak with an *m/z *of 8129 was not different between cases and controls. After splitting our data of the two duplicates into two groups, results were similar. This together with CV's within an acceptable range indicates that our results are robust.

The peaks with an *m/z *of 4276 and 4292 were assumed to represent a fragment of ITIH4 (the 4.3 kDa protein) and its oxidized form. The decrease in intensity of the 4.3 kDa protein in breast cancer cases reported in the study by Li et al. [3] and in that by Mathelin et al. [2] was replicated in our study. The increase in intensity of the 4.3 kDa protein found in the validation study by Li et al. [4] appears to be an exception.

The 8.1 kDa protein, previously identified as C3a_desArgΔ88_[4] was increased in breast cancer cases in both studies by Li et al. [3,4]. This could not be replicated in our study. In our study, no difference in intensity between breast cancer cases and controls was found for this protein, neither as it was in the study by Mathelin et al. [2].

In the three previous studies [2-4], the 8.9 kDa protein, identified as C3a_desArg _was increased in breast cancer cases. This could not be replicated in our study. In our study, this protein was decreased in cases compared to controls, which thus appears to be an exception. Remarkably, a decrease of this protein was also found in two other breast cancer sample-sets analyzed by our group (publication in preparation). Furthermore, it is also remarkable that this protein was the best discriminating protein between breast cancer cases and healthy controls found in this study. The 8941 *m/z *peak found in our study was previously identified by our group as C3a_desArg_, which is thus in agreement with the identity of the 8.9 kDa protein found in the validation study by Li et al. [4]. A summary of the results of the several studies is presented in Table 7.

**Table 7:** Description of the peaks found in the different studies

		**Protein 1**		**Protein 2**	**Protein 3**	
*Li et al. (2002) *[3]	kDa	4.3 ↓		8.1 ↑	8.9 ↑	
	AUC	0.846		0.795	0.934	
	p-value	-		-	-	

*Mathelin et al. *(2005) [2]	*M/z*	4286 ↓	4302 ↓	8129 -	8919 ↑	8961 ↑
	AUC	-	-	-	-	-
	p-value^#^	<0.000	<0.001	0.51	<0.02	<0.001

*Li et al. (2005)*	Da	± 4300* ↑		8116 ↑	8926 ↑	
*Validation study *[4]	AUC	-		0.65	0.71	
	p-value	-		-	-	

*Current study*	*M/z*	4276 ↓	4292 ↓	8129 -	8941 ↓	
	AUC	0.716	0.770	0.510	0.830	
	p-value^†^	<0.0001	<0.0001	0.870	<0.0001	

↓ = Decreased intensity in cases compared to controls, ↑ = Increased intenstiy in cases compared to controls.

*M/z *= Mass to Charge ratio, * Exact mass not reported, ^# ^Test used not reported, ^† ^Mann-Whitney U Test

Li et al. [4] suggested that the inconsistency in regulation of the 4.3 kDa peak (ITIH4 fragment) between their two studies is caused by the instability of this protein. In their first study [3], serum samples from breast cancer patients were collected during a longer time interval than the control samples, whereas samples in the validation study [4] were all collected within the same 2-year window. If the instability of this protein causes further truncation during prolonged storage, this would explain why the intensity of this protein is lower in cases than in controls in Li's first study [3]. In our study, however, as well as in that by Mathelin et al. [2], storage duration of samples did not differ between cases and controls and still in both studies a decrease was found in cases compared to controls. Therefore, the increase of the 4.3 kDa protein found in the validation study by Li et al. [4] cannot be explained by this factor. Also differences in discriminatory power and/or direction of the relation between the several studies for the 8.1 and the 8.9 kDa protein cannot be explained by above mentioned factor.

Another factor that is important for storage is the temperature at which samples are stored. Although in several studies no major differences in serum protein profiles were observed after a storage period of 1–3 months at -20°C, -80°C or liquid nitrogen [10-12], Engwegen et al. [13] found that after a storage duration of 5 months at -20°C compared to -70°C several peaks were significantly increased in intensity. This shows the importance of storing samples at the lowest possible temperature when samples are stored for a prolonged time. Samples analyzed in the studies by Li et al. [3] and Mathelin et al. [2] were stored at -80°C and -20°C, respectively for an unknown time. Samples analyzed in the validation study by Li et al. [4] and in our study were stored at -30°C. Samples of both cases and controls analyzed in the validation study by Li et al. [4] were collected from 2000 on (publication of results in 2005). In our study, samples of both the cases and the controls were stored for less than three and a half years. The exact influence of the differences in storage temperature in combination with storage duration on peak intensities cannot easily be predicted. This is because the influence of these factors on the several proteins is very diverse [14,15]. Over time the intensity of some proteins will decrease because of fragmentation, while the intensities of the fragments of these proteins will increase. However, when these fragments are also very unstable, their intensities could also decrease after a prolonged time. On top of this, some proteins are more vulnerable to degradation than others [14,15]. It is therefore difficult to predict whether these factors have influenced the results of the several studies.

Another, possibly more likely explanation for the discrepant results found in the several studies is differences in pre-analytical sample handling. Several studies have shown that the time between venipuncture and centrifugation as well as the temperature at which samples are held meanwhile is of major influence on protein profiles [10,12-16]. When samples were held for a prolonged time (more than 60 minutes) at room temperature, low mass peaks were generated. These peaks were not formed as long as samples were kept on ice [15]. Previous studies also revealed that some proteins are more vulnerable to sample handling than others [14,15]. Amongst others, fragments of ITIH4 and C3a were found to be increased more than 1.5-fold within 2 hours after venipuncture in samples that were allowed to clot on room temperature [15]. Samples of both cases and controls analyzed in our study were pre-analytically handled identically following a standard protocol. Samples were allowed to clot for 30 minutes at room temperature after which they were centrifuged for 15 minutes at 3000 rpm at room temperature. All blood samples analyzed in the study by Mathelin et al. [2] were allowed to clot at room temperature for a variable duration (30–60 min) after which they were centrifuged for 10 min at 3000 rpm at an unknown temperature. For the studies by Li et al. [3,4] no information about clotting and centrifugation conditions is available. It is also unknown whether samples of cases and controls were pre-analytically handled identically in these studies [3,4]. Therefore we cannot exclude that differences in the sample handling protocols and differences in handling samples of cases and controls in other studies have caused the inconsistent results.

In this study we also examined the relationships between a number of patient and tumor characteristics and peak intensities in order to find possible explanations for the inconsistencies in the literature. Most characteristics appeared to be unrelated to peak intensity, however we did find a relation between the intensity of one of the peaks and menopause status. The previous studies did not provide information on menopause status [3,4] or did not investigate this relation [2].

We also investigated the relations between the intensities of the several peaks and HER2/neu and p53 expression and ER and PR status. Since breast cancer is such a heterogeneous disease, factors causing this heterogeneity, like HER2/neu and p53 expression and ER and PR status [17], should be investigated when searching for potential biomarkers for breast cancer. It is possible that different biomarkers are needed to distinguish different molecular subtypes of breast cancer. No relations between peak intensities and ER/PR status were observed in the studies by Mathelin et al. [2] and by Li et al. [4], neither they were in our study. However, we did find a borderline statistically significant relation between p53 expression and *m/z *8129. It might be possible that differences in ratio of the several molecular subtypes of breast cancer between the studies have led to inconsistencies in the results. However, no information about p53 expression was available in the previous studies [2-4].

## Conclusion

In conclusion, in this study we were able to detect of all three previously reported proteins [2-4]. Most remarkably, two out of the three proteins seemed to be discriminatory but not always in the same direction as in the previous studies. We did not find an immediate explanation for these inconsistencies, but it probably illustrates the susceptibility of proteins to external circumstances. For future studies, more effort should be put into the collection of blood samples of cases and controls with the use of standardized and high quality procedures. Also, a distinction should be made between molecular subtypes of breast cancer in the search for specific tumor markers. The proteins investigated in this study have not yet been proven to be reliable markers for breast cancer.

## Competing interests

The authors declare that they have no competing interests.

## Authors' contributions

AWJW, MCWG and CHG conducted the general design of the study and AWJW and MCWG performed the protein profiling analysis. AWJW and CHG were involved in the data-analysis and drafted the manuscript together with JHB. EJThR included the breast cancer patients. MCWG, DEG and PHMP participated in editing and reviewing of the manuscript. All authors read and approved the final manuscript.

## Pre-publication history

The pre-publication history for this paper can be accessed here:



## Supplementary Material

Additional file 1 **Peaks with statistically significantly different intensities (p < 0.01) in cases compared to controls in order of *m/z*. **An overview of the peaks found in this study that were statistically significantly different in intensity between cases and controls. Click here for file
