# Supplementary material for: Validation of previously identified serum biomarkers for breast cancer with SELDI-TOF MS: a case control study
Source: BMC Med Genomics. 2009 Jan 19;2:4. doi: 10.1186/1755-8794-2-4 (PMC2639617; doi:10.1186/1755-8794-2-4)
Supplement: Additional file 1 — Peaks with statistically significantly different intensities (p < 0.01) in cases compared to controls in order of m/z. An overview of the peaks found in this study that were statistically significantly different in intensity between cases and controls. [file 1755-8794-2-4-S1.doc]

**Additional file 1 - Peaks with statistically significantly different intensities (p<0.01) in cases compared to controls in order of *m/z***

|  | **Breast cancer cases**  (*n*=47) | | **Healthy controls**  (*n*=48) | |  | **Mann-Whitney U test** | **ROC-curve** | |
| --- | --- | --- | --- | --- | --- | --- | --- | --- |
| *M/z* | *Median intensity* | *IQR* | *Median intensity* | *IQR* | *Intensity in cases vs. controls* | *p-value* | *AUC* | *95% CI* |
| 3156 | 11.32 | 7.14-22.94 | 23.11 | 14.03-61.23 | Decreased | <0.0001 | 0.753 | 0.66-0.85 |
| 3270 | 4.33 | 0.84-10.06 | 13.46 | 5.03-21.02 | Decreased | 0.002 | 0.685 | 0.58-0.79 |
| 3965 | 22.13 | 10.42-54.69 | 63.32 | 26.17-80.70 | Decreased | 0.002 | 0.681 | 0.57-0.79 |
| 4276 | 11.04 | 2.10-29.31 | 39.48 | 14.17-77.58 | Decreased | <0.001 | 0.716 | 0.61-0.82 |
| 4292 | 14.54 | 8.52-29.54 | 42.07 | 29.67-63.39 | Decreased | <0.0001 | 0.770 | 0.67-0.87 |
| 4472 | 11.60 | 7.53-15.63 | 16.49 | 12.18-19.92 | Decreased | <0.001 | 0.732 | 0.63-0.83 |
| 5330 | 28.53 | 21.89-50.90 | 20.95 | 11.03-33.15 | Increased | 0.003 | 0.679 | 0.57-0.79 |
| 5896 | 120.51 | 74.47-177.88 | 65.60 | 36.54-107.16 | Increased | <0.001 | 0.735 | 0.64-0.84 |
| 6102 | 15.22 | 8.28-21.20 | 8.56 | 4.18-13.08 | Increased | <0.001 | 0.727 | 0.63-0.83 |
| 7457 | 5.86 | 4.50-6.64 | 4.21 | 2.58-6.31 | Increased | 0.006 | 0.663 | 0.55-0.77 |
| 7754 | 159.15 | 120.82-175.30 | 116.73 | 94.10-145.66 | Increased | 0.002 | 0.687 | 0.58-0.79 |
| 7959 | 16.27 | 12.32-17.73 | 12.17 | 9.44-15.67 | Increased | 0.002 | 0.688 | 0.58-0.80 |
| 8601 | 11.73 | 7.42-15.99 | 20.35 | 12.40-25.34 | Decreased | <0.001 | 0.733 | 0.63-0.83 |
| 8941 | 31.27 | 24.07-44.88 | 73.47 | 48.69-86.72 | Decreased | <0.0001 | 0.830 | 0.75-0.91 |
| 9142 | 2.41 | 0.91-3.68 | 5.35 | 3.39-7.29 | Decreased | <0.0001 | 0.823 | 0.74-0.91 |
| 9280 | 76.29 | 56.37-95.74 | 56.03 | 31.70-75.94 | Increased | <0.001 | 0.707 | 0.60-0.81 |
| 9474 | 10.28 | 7.84-11.94 | 7.50 | 4.02-9.85 | Increased | <0.001 | 0.710 | 0.61-0.81 |
| 10,054 | 1.42 | 0.71-2.50 | 2.47 | 1.64-4.07 | Decreased | 0.003 | 0.676 | 0.57-0.79 |
| 11,720 | 1.66 | 1.30-2.33 | 1.26 | 0.76-1.87 | Increased | 0.002 | 0.684 | 0.58-0.79 |
| 54,214 | 0.16 | 0.13-0.21 | 0.11 | 0.08-0.14 | Increased | <0.001 | 0.730 | 0.63-0.84 |

*M/z* = Mass to Charge ratio, IQR = Inter-Quartile Range, ROC-curve = Receiver Operating Characteristic curve, AUC = Area Under the Curve, 95% CI = 95% Confidence Interval
